# Comparison of Stage 4 and Stage 4s Neuroblastoma Identifies Autophagy-Related Gene and LncRNA Signatures Associated With Prognosis

**DOI:** 10.3389/fonc.2020.01411

**Published:** 2020-08-19

**Authors:** Xinyao Meng, Honglin Li, Erhu Fang, Jiexiong Feng, Xiang Zhao

**Affiliations:** Department of Pediatric Surgery, Tongji Hospital, Tongji Medical College, Huazhong University of Science and Technology, Wuhan, China

**Keywords:** autophagy, neuroblastoma, stage 4s, prognosis, long non-coding RNA

## Abstract

**Background:** The spontaneous regression of neuroblastoma (NB) is most prevalent and well-documented in stage 4s NB patients. However, whether autophagy plays roles in the spontaneous regression of NB is unknown.

**Objective:** This study aimed to identify autophagy-related genes (ARGs) and autophagy-related long non-coding RNAs (lncRNAs) differentially expressed in stage 4 and stage 4s NB and to build prognostic risk signatures on the basis of the ARGs and autophagy-related lncRNAs.

**Methods:** One RNA-sequence (RNA-Seq) dataset (TARGET NBL, *n* = 153) was utilized as discovery cohort, and two microarray datasets (*n* = 498 and *n* = 223) were used as validation cohorts. Differentially expressed ARGs were identified by comparing stage 4s and stage 4 NB samples. An ARG signature risk score and an autophagy-related lncRNA signature risk score were constructed. The receiver operating characteristic (ROC) curve analyses were used to evaluate the survival prediction ability of the two signatures. Gene function annotation and Gene Set Enrichment Analysis (GSEA) were performed to clarify the autophagic biological processes enriched in different risk groups.

**Results:** Nine ARGs were integrated into the ARG signature. Patients in the high-risk group of the ARG signature had significantly poorer overall survival (OS) than patients in the low-risk group. The ROC curves analyses revealed that the ARG signature performed very well in predicting OS [5-year area under the curve (AUC) = 0.81]. Seven autophagy-related lncRNAs were integrated into the autophagy-related lncRNA signature. Patients in the high-risk group of the lncRNA signature had significantly poorer OS than patients in the low-risk group. The ROC curve analyses also revealed that the lncRNA signature performed well in predicting OS (5-year AUC = 0.77). Both the ARG signature and lncRNA signature are independent with other clinical risk factors in the multivariate Cox regression survival analyses. GSEAs revealed that autophagy-related biological processes are enriched in low-risk groups.

**Conclusions:** Autophagy-related genes and lncRNAs are differentially expressed between stage 4 and stage 4s NB. The ARG signature and autophagy-related lncRNA signature successfully stratified NB patients into two risk groups. Autophagy-related biological processes are highly enriched in low-risk NB groups.

## Introduction

Spontaneous regression of cancer has been documented since the 1900s (1). It means that a malignant tumor completely or partially disappears without acceptance of any tumor-associated treatment (1). This interesting and promising biological phenomenon has been observed in various types of cancers (2–6). However, neuroblastoma (NB) is generally considered the most common malignancy in which this phenomenon is most evident and prevalent (3, 7). The spontaneous regression of NB has been validated by several mass screening programs undertaken in different regions of the world including Japan, North America, and Europe or (8–11). This phenomenon is most evident in NB patients with stage 4s disease (3, 12–14). Patients with stage 4s NB usually had a localized primary tumor but with tumors metastasized to the liver, skin, or bone marrow (7). Unlike other metastatic malignancies, NB patients with stage 4s disease generally had a surprisingly good survival outcome, and most of them underwent spontaneous regression even without antitumor treatment (15–17). One study reported a 5-year overall survival (OS) rate of 92% for stage 4s NB patients receiving supportive care or minimal therapy (18). One recent study also reported a complete regression rate of 92% for stage 4s adrenal NB (19).

Spontaneous regression is not restricted to stage 4s NB; it also regularly occurs in infants with localized NB (one study reported a complete regression rate of about 38.6% for localized NB) (20). In fact, it can be observed in any stage of NB if the tumor has biologically favorable histology (7, 15). Because spontaneous regression of NB is most prevalent in patients with stage 4s disease, investigators have been focusing on stage 4s NB as a surrogate to explore the underlining mechanisms responsible for spontaneous regression of NB (7, 12–14). However, the mechanism responsible for the spontaneous regression of NB is still largely unknown.

In recent years, autophagy has been found to play important roles in tumor development and progression (21, 22) and is also involved in NB (23–26). The association between autophagy and spontaneous regression of NB is unknown. Because studies have found that autophagy is associated with NB cell apoptosis and differentiation (23, 24), we wish to know whether autophagy is involved in the process of spontaneous regression.

In this study, as other investigators have done previously (7, 12–14), we also use stage 4s NB as a surrogate. One RNA-sequence (RNA-Seq) datasets (TARGET NBL, *n* = 153) and two microarray datasets (*n* = 498 and *n* = 223) were utilized in this study. Differentially expressed autophagy-related genes (ARGs) were identified by comparing those deceased cases in stage 4 NB and those survived cases in stage 4s NB. As one of our previous study has done before (7), the dead cases in stage 4s were excluded to make it better for serving as surrogates to NBs that underwent spontaneous regression.

Finally, nine differentially expressed and survival-related ARGs were incorporated into the ARG prognostic signature. Seven autophagy-related long non-coding RNAs (lncRNAs) were also identified and incorporated into an autophagy-related lncRNA prognostic signature. The ARG signature and autophagy-related lncRNA signature performed well in predicting OS of NB patients. Gene Ontology (GO) function annotation and Gene Set Enrichment Analysis (GSEA) revealed that autophagy-related biological processes were significantly enriched in the low-risk groups, whereas no autophagy gene set was identified in the high-risk groups. These results reveal that autophagy tends to play tumor-suppressive roles in NB and might be associated with the spontaneous regression of NB.

## Materials and Methods

### Neuroblastoma Dataset Processing

The processed data of the RNA-Seq dataset (TARGET NBL, *n* = 153) were downloaded from National Cancer Institute GDC Data Portal. The original data of the TARGET NBL obtained from GDC Data Portal have a total of 161 samples. Two paired duplicated samples were identified; the gene express values in the duplicated sample are the same too, and thus, we excluded these duplicated samples during the analysis. We also identified that six paired samples are from the same patients. The clinical information for these paired samples is the same, whereas one sample was obtained from the original tumor and the other one sample was obtained from recurrent tumor. In order to reduce confounding factors, we also excluded those six recurrent tumor samples and kept their corresponding primary tumor samples only. Finally, 153 samples were kept for the analyses, with 73 stage 4 NB samples from patients who died and 19 stage 4s samples from patients who survived during the follow-up.

The processed data of the Agilent microarray datasets GSE49710 (*n* = 498) were obtained from Gene Expression Omnibus (GEO) database. The processed data of the Agilent microarray datasets E-MTAB-8248 (*n* = 223) were obtained from ArrayExpress database. The genes express levels in the three datasets were already processed and log2 transformed. The clinical characteristics of the patients in these three datasets were also obtained and are shown in Supplementary Table 1. The RNA-Seq dataset (termed as cohort 1) was used as the discovery cohort. The microarray datasets GSE49710 (termed as cohort 2) and E-MTAB-8248 (termed as cohort 3) were used as the validation cohorts. The Agilent microarray probes IDs were firstly annotated using the platform GPL16876 (Agilent-020382 Human Custom Microarray 44k); then, the probes IDs were re-annotated by their GenBank Accession number in order to renew the annotation. Finally, in order keep consistency over the three datasets, the Ensemble ID in the three datasets was transformed into gene symbols according to GRCh38.p12, and the background of the three datasets was also intersected normalized by R package “sva.” If multiple probes mapped to one same gene, the average level of the expression value will be used. The online platform of cBio Cancer Genomics Portal (cBioportal) (http://www.cbioportal.org/) was utilized to analyze the genomic alteration (mutation and copy number alteration) of the identified genes (27).

### Extraction of Differentially Expressed Autophagy-Related Genes

ARGs were extracted from Human Autophagy Database (https://www.autophagy.lu/), with a total of 232 ARGs. Differential expression analyses were performed by “limma” package using the R (version 3.6.2) software in cohort 1. Genes with false discovery rate (FDR) < 0.05 and |log2FoldChange| > 0.5 were extracted as differentially expressed genes. LncRNAs correlated (Pearson correlation threshold ≥ 0.5) with ARGs were extracted as autophagy-related lncRNAs. Only those lncRNAs matching the GENCODE annotation of lncRNA (release 31, GRCh38.p12) were selected.

### Construction of the Autophagy-Related Prognostic Signatures

Univariate Cox proportional hazards regression analyses were performed to identify those ARGs associated with OS in the entire cohort 1. A *p* ≤ 0.05 was considered statistically significant. Those survival-related ARGs were put into the Cox proportional hazards model survival analysis with least absolute shrinkage and selection operator (LASSO) penalty to eliminate false positives owing to over-fitting (28). Finally, the autophagy-related prognostic signature was constructed by weighting the Cox regression coefficients for each gene to calculate a risk score for every patient. The median value was used as the cutoff value, and the patients were classified as low risk and high risk accordingly. The same formula was applied to the validation cohorts, and the same cutoff value was used to divide the patients into two risk groups. Autophagy-related lncRNA signature was constructed by the same method. Those autophagy-related lncRNAs associated with OS were put into LASSO Cox model regression analyses. The autophagy-related lncRNA signature was constructed by the same method.

### Function Annotation and Gene Set Enrichment Analysis

Differentially expressed ARGs that associated with OS were put into GO functional annotation. The GO function annotation was first performed by R software using “BiocManager” package of “clusterProfiler,” “org.Hs.eg.db,” and “enrichplot.” Then the circle plot of GO function annotation was generated by R software using package “GOplot.” Functional annotation with a *p* < 0.05 was considered statistically significant. GSEA comparing low-risk group and high-risk group was performed by GSEA software (version 4.0.03). An FDR *q*-value < 0.25 and a nominal *p* < 0.05 were considered statistically significant for GSEAs.

### Statistical Analysis

The univariate and multivariate Cox survival regression analyses were calculated by the R package “survival.” The LASSO Cox survival analyses were performed by the R package “glmnet,” and 1,000-fold cross-validation was used. The Kaplan–Meier survival curves were constructed by R software or GraphPad Prism 5, and the statistical significance was estimated by the two-sided log-rank test. The time-dependent receiver operating characteristic (ROC) curves and area under the curve (AUC) analyses were performed to evaluate the predictive performance of the prognostic signatures and performed by the R package “time ROC.” Nomograms were generated by R package “rms,” and Harrell's concordance index (*C*-index) was calculated to evaluate the discriminatory ability. Volcano plot was plotted by the R package “ggplot2.” Heat maps were generated by the R package “pheatmap.” The Pearson correlation matrix was generated by the R package “corrplot.” The alluvial diagrams were generated by the R package “ggalluvial.” The R software version 3.6.2 was utilized in this study for the statistical analyses. A *p* < 0.05 was considered statistically significant, and all statistical tests were two-sided.

## Results

### Identification of Differentially Expressed and Survival-Related Autophagy-Related Genes

**Figure 1:**
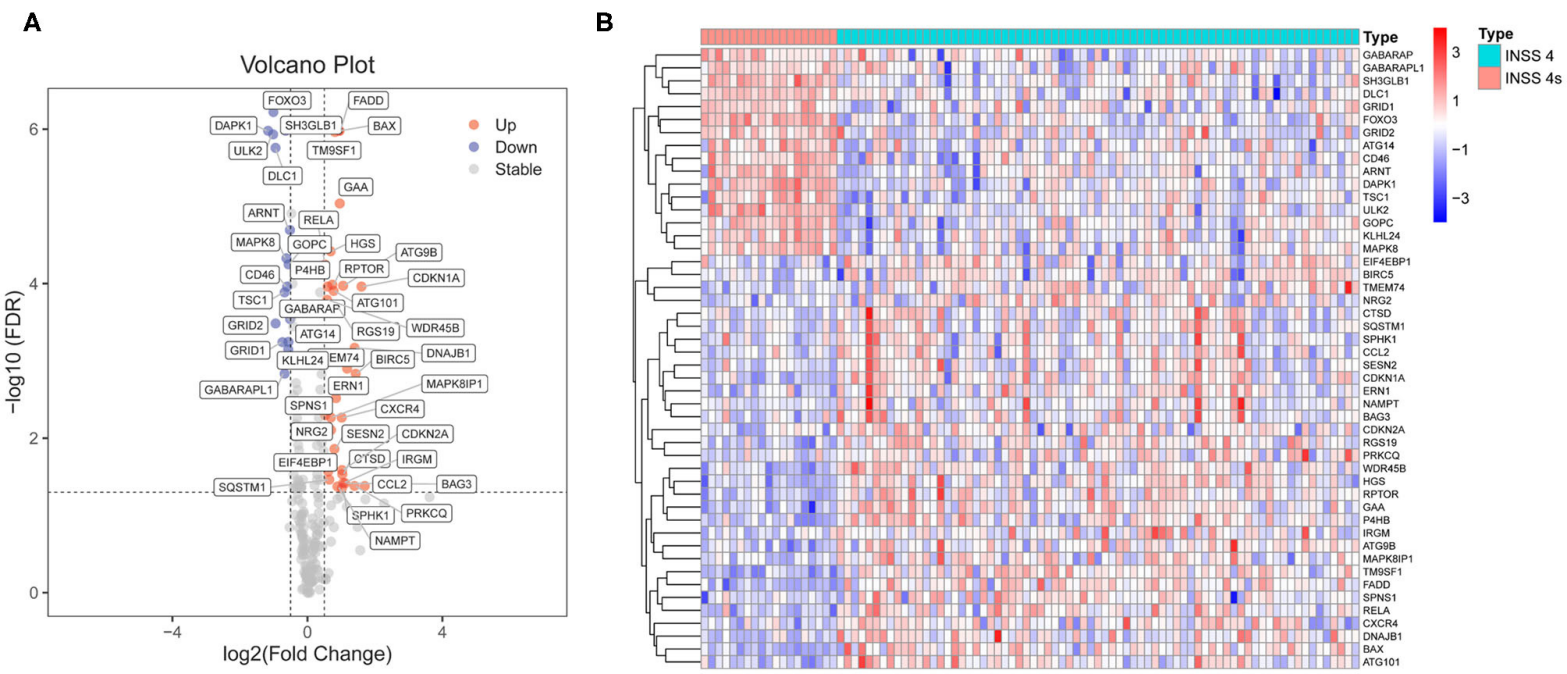
Identification of differentially expressed autophagy-related genes (ARGs) between stage 4s and stage 4 neuroblastoma (NB). **(A)** Volcano plot shows the differentially expressed 48 ARGs in cohort 1. **(B)** The heat map shows the expression values of the identified 48 ARGs in cohort 1.

Differential expression analyses were performed on the RNA-Seq datasets (cohort 1, *n* = 153). Cohort 1 contains 125 stage 4 NB samples and 21 stage 4s NB samples. A total of 48 ARGs were found to be differentially expressed between those stage 4 cases who died during follow-up (*n* = 73) and those stage 4s cases who survived during follow-up (*n* = 19). Thirty-two ARGs were up-regulated in stage 4 NB samples, whereas 16 ARGs were up-regulated in stage 4s NB samples (Figures 1A,B).

Univariate Cox proportional model survival analyses revealed that 19 ARGs were significantly (*p* < 0.05) associated with OS in the entire cohort 1 (Supplementary Figure 1A). Twelve ARGs were up-regulated in stage 4s NB samples and associated with good survival, whereas seven ARGs were up-regulated in stage 4 NB samples and associated with bad survival.

### Construction and Validation of Autophagy-Related Gene Prognostic Signature

**Figure 2:**
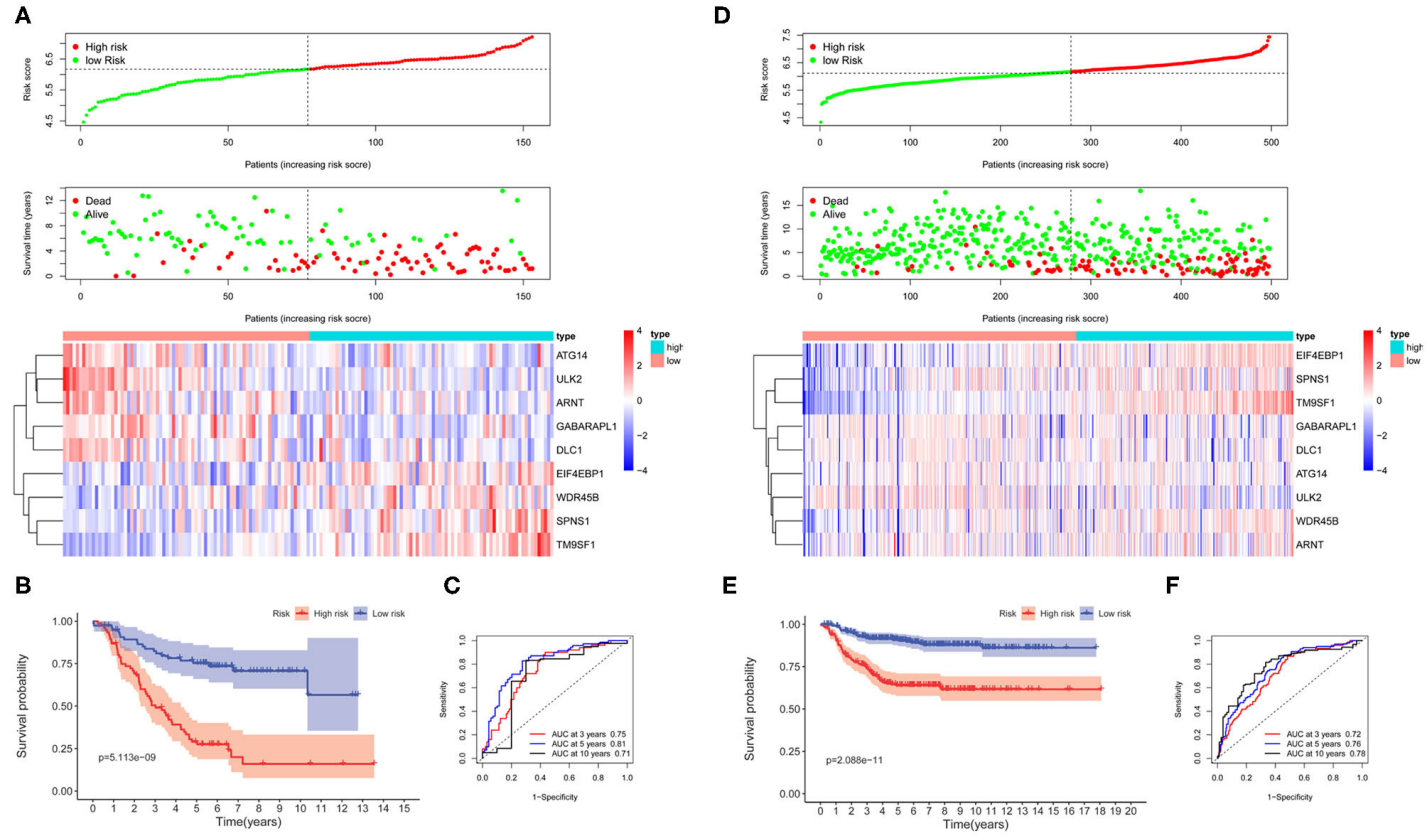
The autophagy-related genes (ARGs) prognostic signature for neuroblastoma. **(A)** The distribution of risk scores, survival status of each patient, and heat map of ARG expression pattern in cohort 1. **(B)** Kaplan–Meier survival curve for overall survival (OS) of patients in the low-risk group and high-risk group for cohort 1. **(C)** Time-dependent receiver operating characteristic (ROC) curves for the prognostic value of the ARG signature in cohort 1. **(D)** The distribution of risk scores, survival status of each patient, and heat map of ARG expression pattern in cohort 2. **(E)** Kaplan–Meier survival curve for OS of patients in the low-risk group and high-risk group for cohort 2. **(F)** Time-dependent ROC curves for the prognostic value of the ARG signature in cohort 2.

The survival-related ARGs were put into LASSO Cox survival analysis to eliminate false positives (Supplementary Figures 1B,C). The 1 – SE criterion revealed only one gene (TM9SF1) in the model; thus, the “lambda.min” criterion was used to select the minimum lambda value (λ = 0.0677). Finally, nine ARGs (Supplementary Table 2) were selected and incorporated into the ARG signature risk score. The risk scores were calculated for each patient as follows: risk score = 0.1248 ^*^ SPNS1 + 0.6746 ^*^ TM9SF1 + 0.0145 ^*^ WDR45B + 0.0088 ^*^ EIF4EBP1 – 0.0012 ^*^ GABARAPL1 – 0.0649 ^*^ ATG14 – 0.0810 ^*^ ULK2 – 0.0165 ^*^ DLC1 – 0.0269 ^*^ ARNT. The median value was used as the cutoff value, and the entire cohort 1 was classified into two risk groups accordingly. The risk distribution, survival status, and gene expression pattern are shown in Figure 2A. The scatter plot (Figure 2A) shows that most of the patients in the high-risk group died and most of the patients in the low-risk group survived during the 15-year follow-up. The heat map (Figure 2A) shows that five ARGs were highly expressed in the low-risk group, whereas four ARGs were highly expressed in the high-risk group. Kaplan–Meier plots show that patients in the high-risk group have a significantly poorer OS than those in the low-risk group (Figure 2B). The 3-, 5-, and 10-year OS rates for the patients in high-risk group were 50.42, 28.97, and 16.01%, respectively, whereas the 3-, 5-, and 10-year OS rates for patients in low-risk group were 82.38, 75.28, and 70.66%, respectively. Time-dependent ROC curves reveal that the ARG signature has good performance in predicting OS in cohort 1, whereas the AUC at 3-, 5-, and 10-years were 0.75, 0.81, and 0.71, respectively (Figure 2C).

To corroborate the prognostic significance, the ARG signature was tested in cohort 2 (*n* = 498) and cohort 3 (*n* = 223) for validation using the same risk score formula. According to the same cut-off value as cohort 1, the validation cohorts were divided into two risk groups. The risk distribution, survival status, and gene expression pattern for cohort 2 are shown in Figure 2D. Kaplan–Meier plots show that patients in the high-risk group have a significantly poorer OS than those in the low-risk group in cohort 2 (Figure 2E). Time-dependent ROC curves reveal that the ARG signature has good performance in predicting OS in cohort 2, whereas the AUC at 3-, 5-, and 10-years was 0.72, 0.76, and 0.78, respectively (Figure 2F). Consistent with cohort 1 and cohort 2, the validation in cohort 3 shows similar results (Supplementary Figure 2).

### Survival Analysis for the Autophagy-Related Gene Prognostic Signature

**Figure 3:**
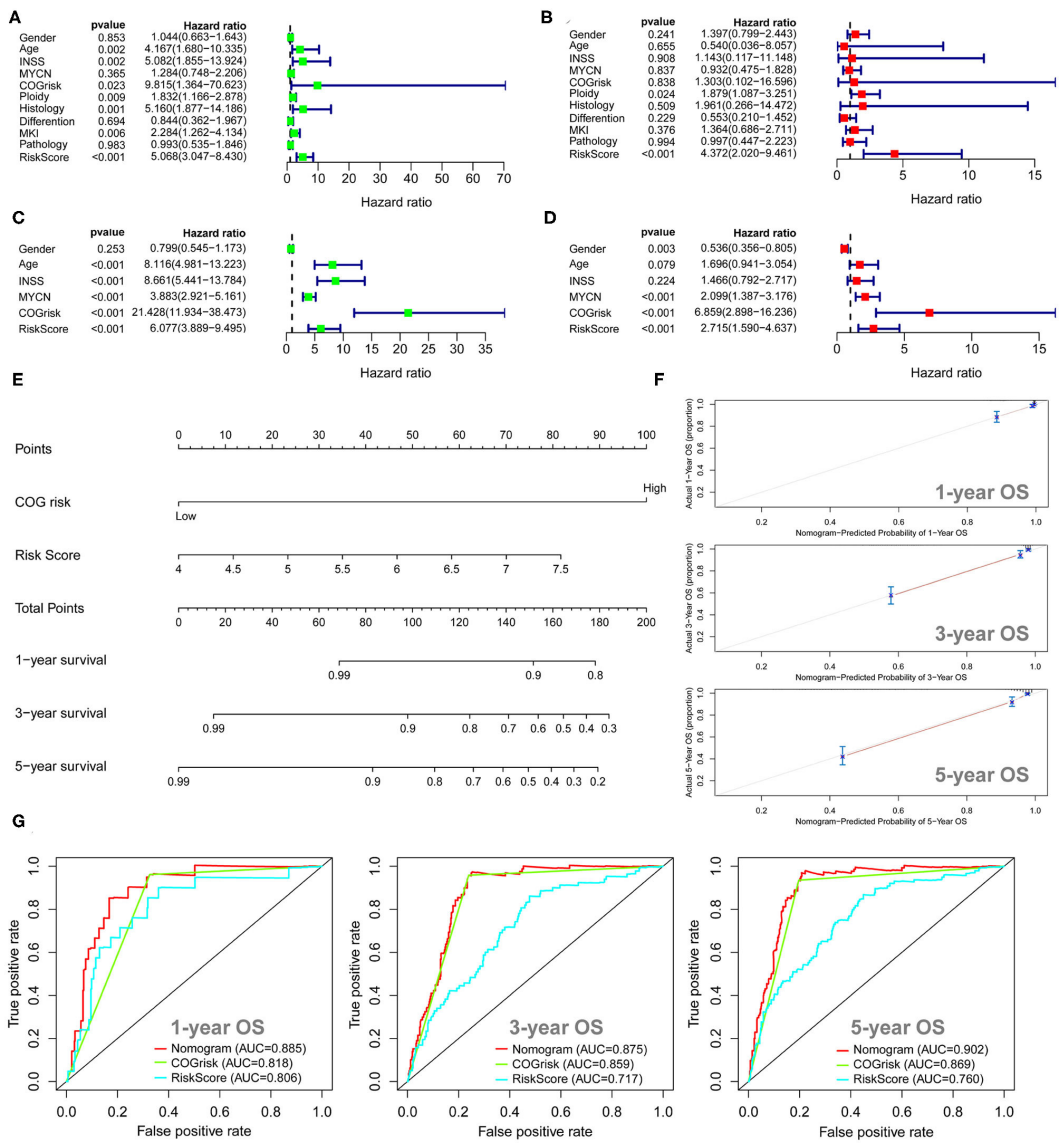
Univariate and multivariate survival analyses of the autophagy-related gene (ARG) signature. **(A)** Univariate survival analysis of the ARG signature and other clinical risk factors in cohort 1. **(B)** Multivariate survival analysis of the ARG signature and other clinical risk factors in cohort 1. **(C)** Univariate survival analysis of the ARG signature and other clinical risk factors in cohort 2. **(D)** Multivariate survival analysis of the ARG signature and other clinical risk factors in cohort 2. **(E)** The nomogram model for prediction of overall survival in cohort 2. **(F)** The 1-, 3-, and 5-year calibration curves for the nomogram. **(G)** The 1-, 3-, and 5-year ROC curve analyses for the nomogram.

The univariate Cox regression survival analyses for the ARG signature risk score and other clinical risk factors in the entire cohort 1 are shown in Figure 3A. The ARG signature risk score is significantly associated with OS [hazard ration (HR) = 5.068; 95%CI: 3.047–8.430; *p* < 0.001] in the univariate survival analysis. Multivariate Cox survival analyses including gender (female vs. male), age status (<18 vs. ≥18 months), International Neuroblastoma Staging System (INSS) stage (INSS 2/3/4S vs. INSS 4), MYCN amplification (non-amplified vs. amplified), Children's Oncology Group (COG) risk status (low risk vs. high risk), ploidy (hyperploid vs. diploid), histology type (favorable vs. unfavorable), differentiation (differentiating vs. poorly differentiated), mitosis-karyorrhexis index (MKI) (low/intermediate vs. high), and pathology subtype (ganglioneuroblastoma vs. NB) as covariates were performed to evaluate the independent prognostic role of the ARG signature (Figure 3B). In cohort 1, only the ARG signature (HR = 4.372; 95%CI: 2.020–9.461, *p* < 0.001) and ploidy (HR = 1.897; 95%CI: 1.087–3.251; *p* = 0.024) were independently associated with OS (Figure 3B). The univariate and multivariate Cox regression survival analyses for the ARG signature and other clinical risk factors in cohort 2 are shown in Figures 3C,D. The ARG signature risk score is significantly associated with OS in cohort 2 by both univariate model (HR = 6.077; 95%CI: 3.889–9.495; *p* < 0.001) and multivariate model (HR = 2.715; 95%CI: 1.590–4.637; *p* < 0.001). Because the COG risk group classification already considered age, MYCN amplification, and INSS stage into its risk classification system, we built a nomogram incorporating only the COG risk classification and the ARG signature risk score for prediction of OS on the basis of the largest cohort (cohort 2, *n* = 498) (Figure 3E). As is shown in the nomogram (Figure 3E), COG low risk was denoted as 0 point, whereas COG high risk was denoted as 100 points. As for the ARG signature risk score in the nomogram, a risk score of 4 was denoted as 0 point, and a risk score of 7.5 was denoted as 82 points. The risk scores between 4 and 7.5 were assigned correspondingly between 0 and 82 points and could be calculated as follows: point = (risk score – 4) ^*^ (82/3.5). The total points for the patients were calculated by combining the points for COG risk and the points for the ARG risk score, and the corresponding predicted survival probability are shown below.

The *C*-index for the nomogram was 0.84 (95%CI: 0.81–0.87), indicating a high level of accuracy. The 1-, 3-, and 5-year calibrate curves for the nomogram all revealed that the predicted OS was very close to the actual OS (Figure 3F). The ROC curve analyses reveal that the AUC values at 1-, 3-, and 5-years for the nomogram were higher than the AUC values at 1-, 3-, and 5-years for the COG risk, respectively (Figure 3G), indicating that the prognostic role of the nomogram is more accurate than the COG risk classification alone.

### Prognostic Role of the Autophagy-Related Gene Signature Within Clinical Subgroups

**Figure 4:**
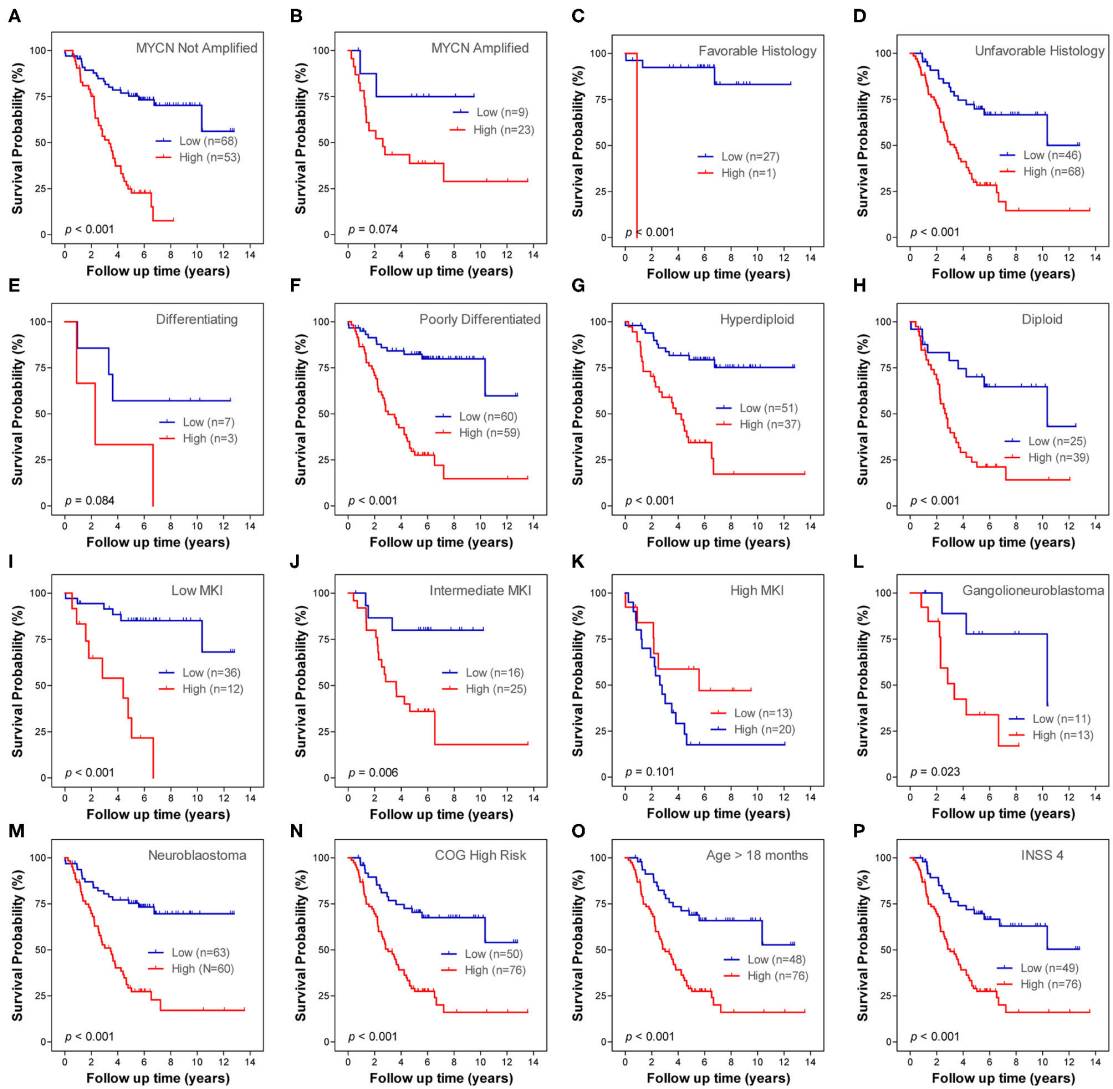
Kaplan–Meier plots show the prognostic role of the autophagy-related gene (ARG) signature for overall survival in different subgroups of cohort 1. **(A)** MYCN not amplified. **(B)** MYCN amplified. **(C)** Favorable histology. **(D)** Unfavorable histology. **(E)** Differencing. **(F)** Poorly differentiated. **(G)** Hyperdiploid. **(H)** Diploid. **(I)** Low mitosis-karyorrhexis index (MKI). **(J)** Intermediate MKI. **(K)** High MKI. **(L)** Ganglioneuroblastoma. **(M)** Neuroblastoma. **(N)** Children's Oncology Group (COG) high risk. **(O)** Age > 18 months. **(P)** International Neuroblastoma Staging System (INSS) stage 4. The *p-*values were obtained using a Mantel log-rank test (two-sided).

Stratification survival analysis was performed to evaluate the prediction ability of the ARG signature in different clinical subgroups. The subgroups were classified based on MYCN amplification status, histology subtype, differentiation status, ploidy status, MKI status, pathology subtype, COG risk status, age status, and INSS stage. Within each subgroup, patients were stratified into low-risk group and high-risk group on the basis of the same cut-off value from the entire cohort 1. In the MYCN non-amplified subgroup, patients in the high-risk group had a significantly worse OS than patients in the low-risk group (Figure 4A), whereas the ARG signature failed to stratify patients in the MYCN amplified subgroup into two risk groups (Figure 4B). In both of the histology subtype (favorable and unfavorable), patients in the high-risk group had significantly worse OS than patients in the low-risk group (Figures 4C,D). In the differentiating subgroup, the ARG signature failed to successfully stratify patients into two risk groups (Figure 4E), whereas in the poorly differentiated subgroup, patients in the high-risk group had significantly worse OS than patients in the low-risk group (Figure 4F). In both of the ploidy subtype (hyperdiploid and diploid), patients in the high-risk group had significantly worse OS than patients in the low-risk group (Figures 4G,H). In both the low MKI subgroup and intermediate MKI subgroup, patients with a high-risk score had a significantly worse OS than patients with a low-risk score (Figures 4I,J); however, the ARG signature failed to stratify patients in the high MKI subgroup into two risk groups (Figure 4K). In both of the ganglioneuroblastoma subgroup and NB subgroup, patients with a high-risk score had a significantly worse OS than those with a low-risk score (Figures 4L,M). All patients in the COG low-risk subgroup were classified as ARG low-risk group; thus, the Kaplan–Meier plot was not constructed, whereas the ARG risk score significantly stratify patients in the COG high-risk subgroup into two risk groups for OS (Figure 4N). All patients with diagnosis age < 18 months were classified as ARG low-risk group; thus, the Kaplan–Meier plot was not constructed, whereas the ARG risk score significantly stratify patients in the age > 18 month subgroup into two risk groups for OS (Figure 4O). All patients in stage 4s were classified as ARG low-risk group; thus, the Kaplan–Meier plot was not constructed, whereas the ARG risk score significantly stratifies patients in the stage 4 subgroup into two risk groups for OS (Figure 4P). There is only one patient classified as stage 2, six patients classified as stage 3, and no patients classified as stage 1 in cohort 1. Thus, we did not conduct subgroup analysis for stage 1, stage 2, and stage 3.

### Construction and Validation of Autophagy-Related LncRNA Prognostic Signature

**Figure 5:**
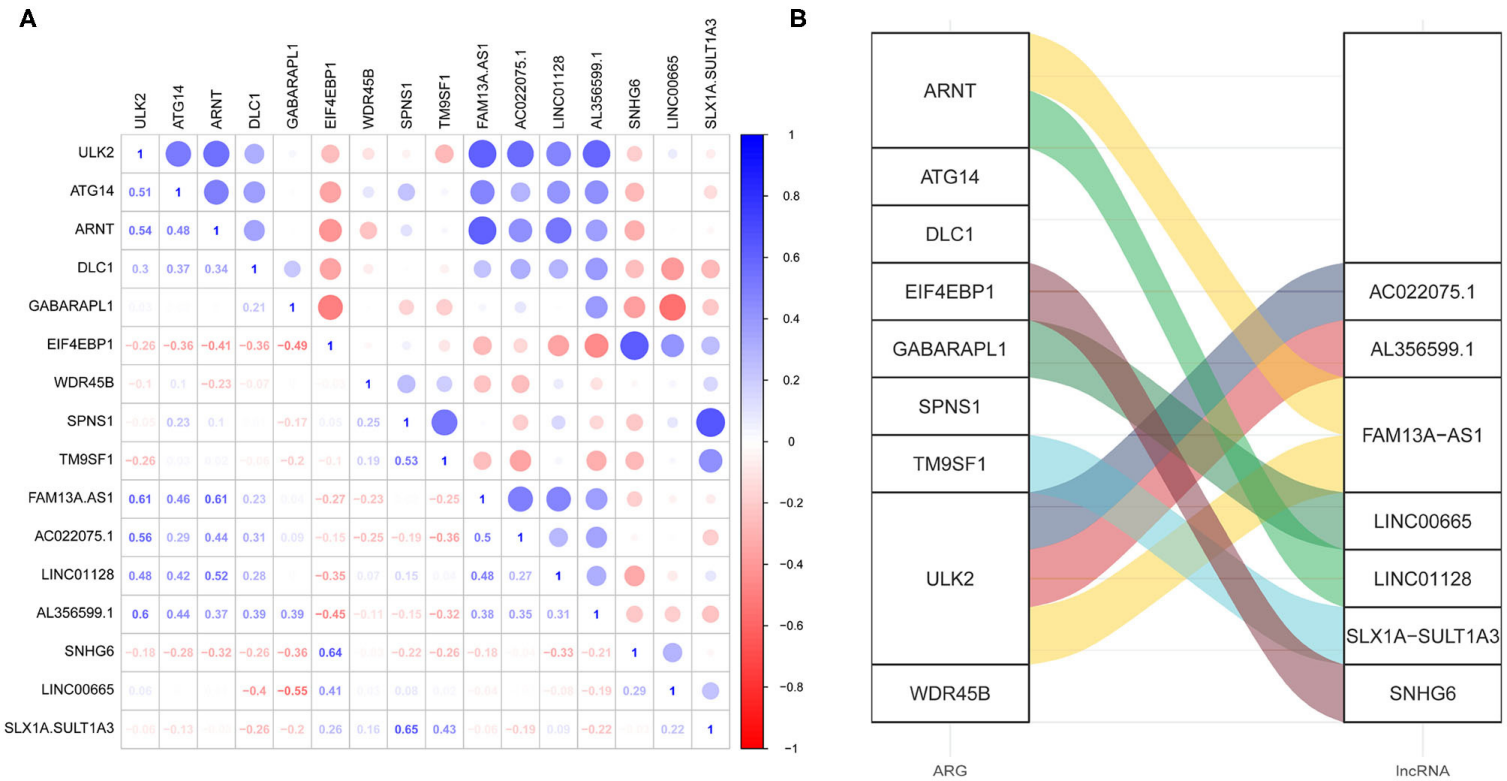
The correlation between the autophagy-related genes (ARGs) and the autophagy-related long non-coding RNAs (lncRNAs). **(A)** The correlation matrix showing the Pearson correlation coefficients. **(B)** The alluvial diagram showing the correlation between the ARGs and the lncRNAs, which has a coefficient ≥ 5.

The lncRNAs correlated (Pearson correlation threshold ≥ 0.5) with the nine ARGs in the ARG signature were extracted as autophagy-related lncRNAs. A total of 562 autophagy-related lncRNAs were identified in cohort 1. However, only 18 autophagy-related lncRNA were shown to be significantly associated with OS by the univariate Cox survival analysis (Supplementary Figure 3A). The survival-related lncRNAs were put into LASSO Cox survival analysis to eliminate false positives. The 1 – SE criterion revealed no gene in the model; thus, the minimum lambda value was selected (λ = 0.0419) (Supplementary Figures 3B,C). Finally, seven autophagy-related lncRNAs (Supplementary Table 3) were selected and incorporated into the lncRNA signature risk score. The correlation between these seven lncRNAs and the ARGs is shown in Figures 5A,B. Figure 5A shows their Pearson correlation coefficients, whereas Figure 5B shows the links between the ARGs and lncRNAs, which have a coefficient ≥ 0.5.

**Figure 6:**
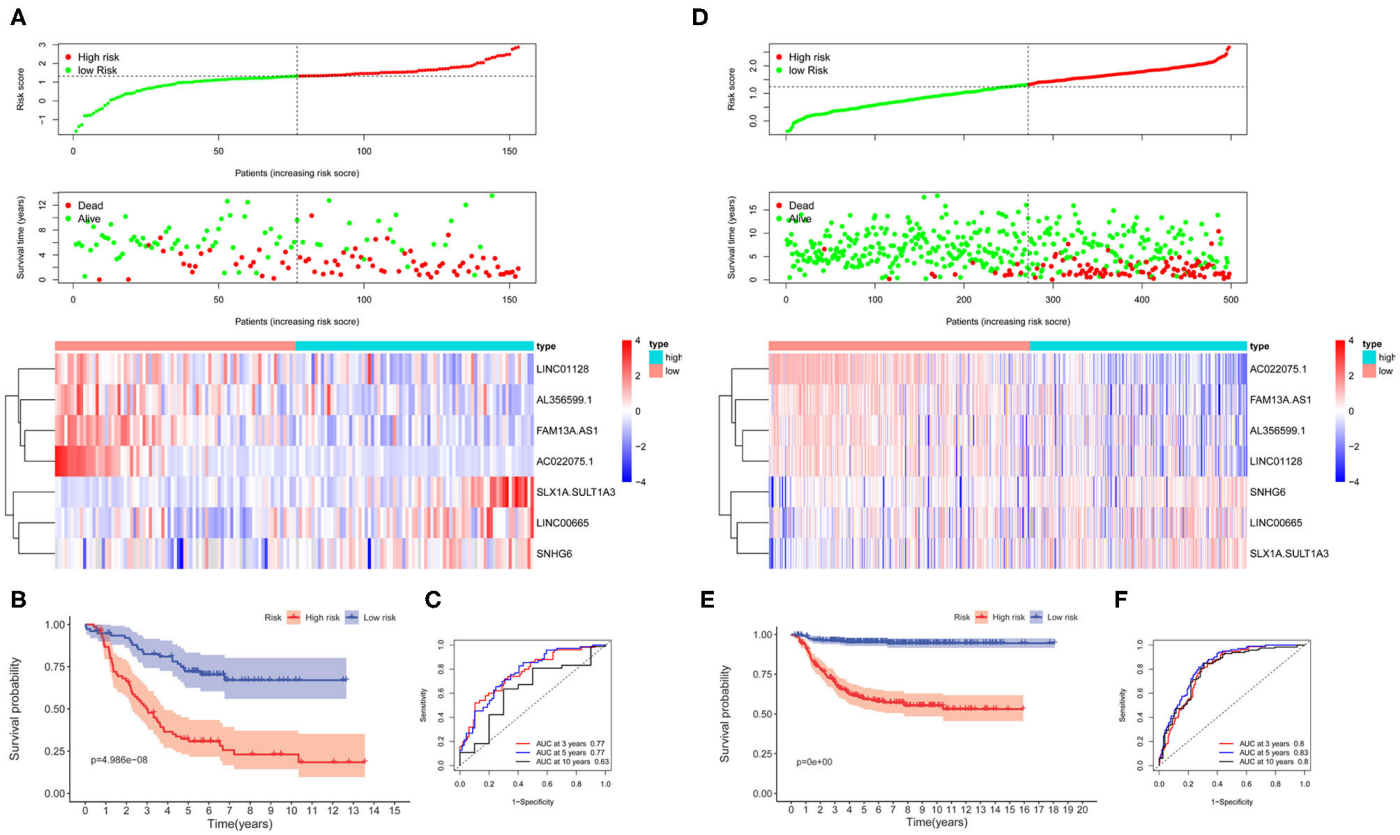
The autophagy-related long non-coding RNA (lncRNA) signature risk score for neuroblastoma. **(A)** The distribution of risk scores, survival status of each patient, and heat map of lncRNA expression pattern in cohort 1. **(B)** Kaplan–Meier survival curve for overall survival (OS) of patients in the low-risk group and high-risk group for cohort 1. **(C)** Time-dependent receiver operating characteristic (ROC) curves for the prognostic value of the lncRNA signature in cohort 1. **(D)** The distribution of risk scores, survival status of each patient, and heat map of lncRNAs expression pattern in cohort 2. **(E)** Kaplan–Meier survival curve for OS of patients in the low-risk group and high-risk group for cohort 2. **(F)** Time-dependent ROC curves for the prognostic value of the lncRNA signature in cohort 2.

The lncRNA signature risk scores were calculated for each patient as follows: risk score = 0.4820 ^*^ SLX1A – SULT1A3 + 0.0578 ^*^ LINC00665 + 0.0050 ^*^ SNH6 – 0.1992 ^*^ FAM13A-AS1 – 0.1984 ^*^ AC022075.1 – 0.0273 ^*^ LINC01228 – 0.0084 ^*^ AL356599.1. The median value was used as the cutoff value, and the entire cohort 1 was classified into two risk groups accordingly. The risk distribution, survival status, and gene expression pattern are shown in Figure 6A. The scatter plot (Figure 6A) shows that most of the patients in the high-risk group died and that most of the patients in the low-risk group survived during the 15-year follow-up. The heat map (Figure 6A) shows that four lncRNAs were highly expressed in the low-risk group whereas three lncRNAs were highly expressed in the high-risk group. Kaplan–Meier plots show that patients in the high-risk score group have a significantly worse OS than those in the low-risk score group (Figure 6B). The 3-, 5-, and 10-year OS rates for the patients in high-risk group were 50.39, 34.24, and 32.03%, respectively, whereas, the 3-, 5-, and 10-year OS rates for patients in low-risk group were 82.42, 72.16, and 66.96%, respectively. Time-dependent ROC curves reveal that the lncRNA signature has good performance in predicting OS in cohort 1, whereas the AUC at 3-, 5-, and 10-years was 0.77, 0.77, and 0.63, respectively (Figure 6C).

The lncRNA signature was tested in cohort 2 (*n* = 498) and cohort 3 (*n* = 223) for validation using the same risk score formula. According to the same cut-off value as cohort 1, the validation cohorts were divided into two risk groups. The risk distribution, survival status, and gene expression pattern for cohort 2 are shown in Figure 6D. Kaplan–Meier plots show that patients in the high-risk score group have a significantly poorer OS than those in the low-risk score group in cohort 2 (Figure 6E). Time-dependent ROC curves reveal that the lncRNA signature has good performance in predicting OS in cohort 2, whereas the AUC at 3-, 5-, and 10-years was 0.8, 0.83, and 0.8, respectively (Figure 6F). Consistent with cohort 1 and cohort 2, the validation in cohort 3 shows similar results (Supplementary Figure 4).

### Survival Analysis for the Autophagy-Related LncRNA Prognostic Signature

**Figure 7:**
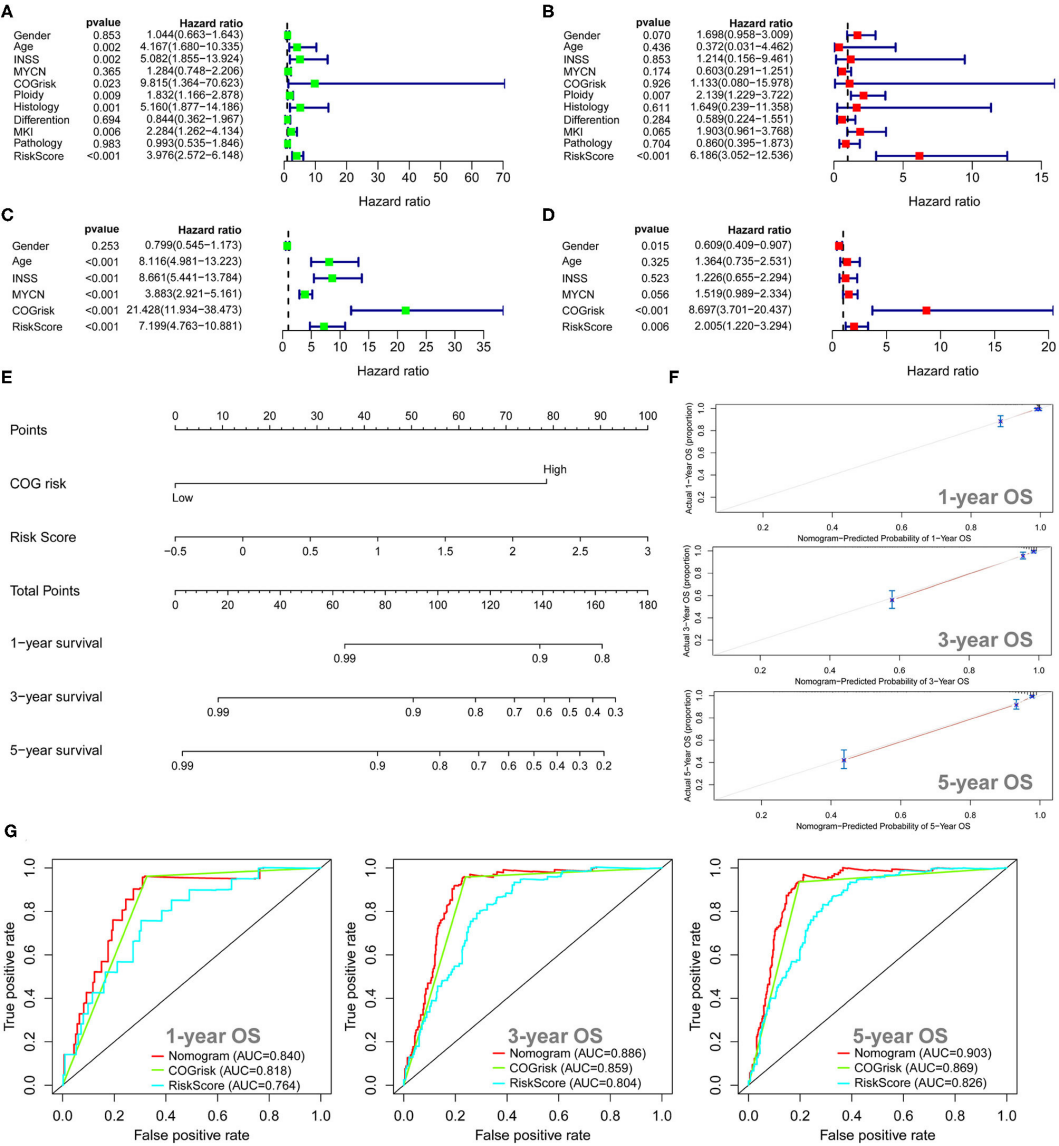
Univariate and multivariate survival analyses for the autophagy-related long non-coding RNA (lncRNA) signature. **(A)** Univariate survival analysis of the lncRNA signature and other clinical risk factors in cohort 1. **(B)** Multivariate survival analysis of the lncRNA signature and other clinical risk factors in cohort 1. **(C)** Univariate survival analysis of the lncRNA signature and other clinical risk factors in cohort 2. **(D)** Multivariate survival analysis of the lncRNA signature and other clinical risk factors in cohort 2. **(E)** The nomogram model for prediction of overall survival in cohort 2. **(F)** The 1-, 3-, and 5-year calibration curves for the nomogram. **(G)** The 1-, 3-, and 5-year receiver operating characteristic (ROC) curve analyses for the nomogram.

The univariate Cox regression survival analyses for the lncRNA signature risk sore and other clinical risk factors in cohort 1 are shown in Figure 7A. The lncRNA signature risk score is significantly associated with OS (HR = 3.976; 95%CI: 2.572–6.148; *p* < 0.001) in the univariate survival analysis. Multivariate Cox survival analyses including gender (female vs. male), age status (<18 vs. ≥18 months), INSS stage (INSS 2/3/4S vs. INSS 4), MYCN amplification (non-amplified vs. amplified), COG risk status (low risk vs. high risk), ploidy (hyperploid vs. diploid), histology type (favorable vs. unfavorable), differentiation (differentiating vs. poorly differentiated), MKI (low/intermediate vs. high), and pathology subtype (ganglioneuroblastoma vs. NB) as covariates were performed to evaluate the independent prognostic role of the lncRNA signature (Figure 7B). In cohort 1, only the lncRNA signature (HR = 6.186; 95%CI: 3.052–12.536, *p* < 0.001) and ploidy (HR = 2.139; 95%CI: 1.229–3.772; *p* = 0.007) were independently associated with OS (Figure 7B). The univariate and multivariate Cox regression survival analyses for the lncRNA signature risk sore and other clinical risk factors in cohort 2 are shown in Figures 7C,D. The lncRNA signature risk score is significantly associated with OS in cohort 2 by both univariate model (HR = 7.199; 95%CI: 4.763–10.881; *p* < 0.001) and multivariate model (HR = 2.005; 95%CI: 1.220–3.294; *p* = 0.006). We also built a nomogram incorporating the COG risk classification and the lncRNA signature risk score for prediction of OS on the basis of the largest cohort (cohort 2, *n* = 498) (Figure 7E). As is shown in the nomogram (Figure 7E), COG low risk was denoted as 0 point, whereas COG high risk was denoted as 79 points. As for the lncRNA signature risk score in the nomogram, a risk score of −0.5 was denoted as 0 point, and a risk score of 3 was denoted as 100 points. The risk scores between −0.5 and 3 were assigned correspondingly between 0 and 100 points and could be calculated as follows: points = (risk score + 0.5) ^*^ (100/3.5). The total points for the patients were calculated by combining the points for COG risk and the points for the lncRNA risk score. The corresponding predicted survival probability is shown below.

The *C*-index for the nomogram was 0.85 (95%CI: 0.81–0.88), indicating a high level of accuracy. The 1-, 3-, and 5-year calibrate curves for the nomogram all revealed that the predicted OS was very close to the actual OS (Figure 7F). The ROC curves analyses revealed that the AUC values at 1-, 3-, and 5-years for the nomogram were higher than the AUC values at 1-, 3-, and 5-years for the COG risk, respectively (Figure 7G), indicating that the prognostic role of the nomogram is more accurate than the COG risk classification alone.

### Prognostic Role of the Autophagy-Related LncRNA Signature Within Clinical Subgroups

**Figure 8:**
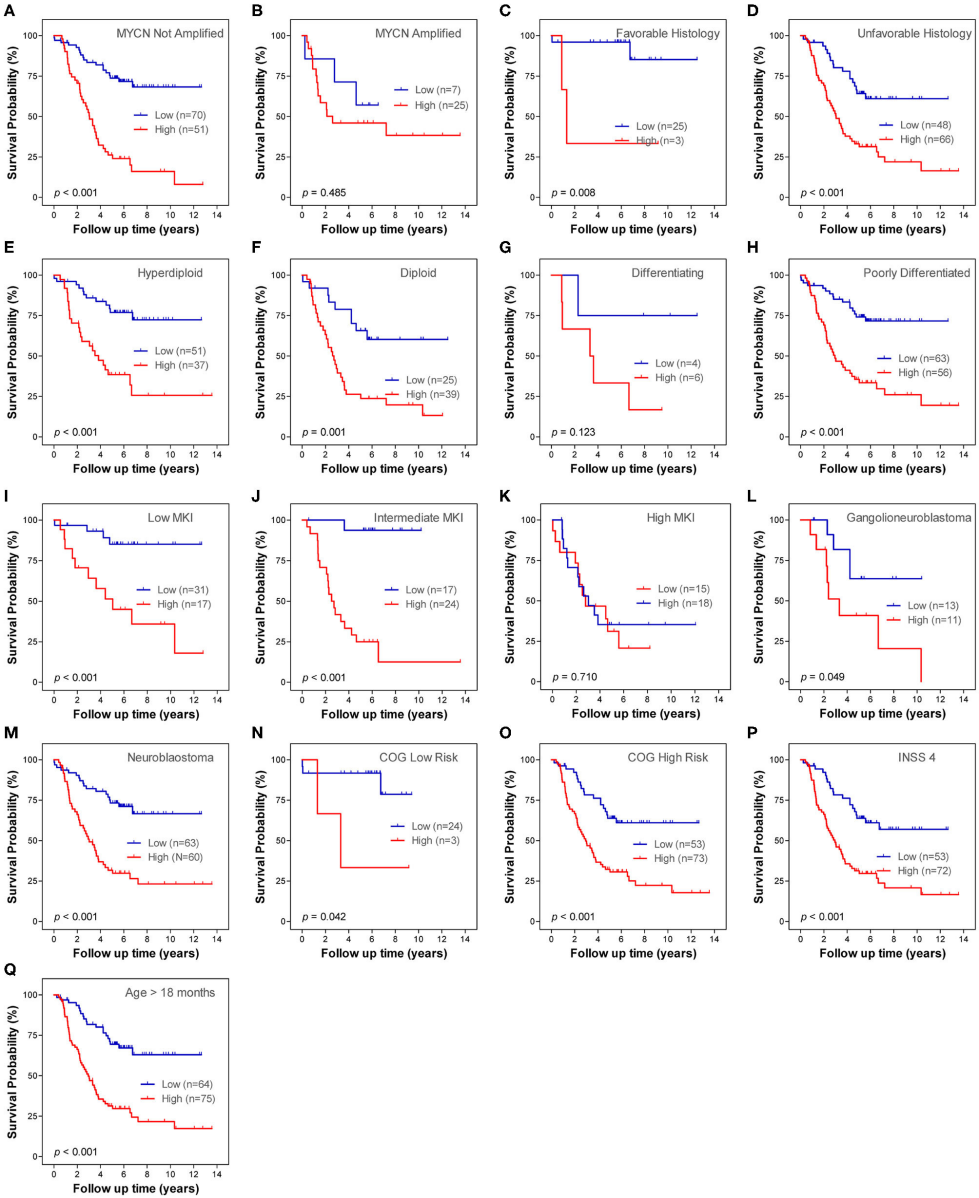
Kaplan–Meier plots show the prognostic role of the long non-coding RNA (lncRNA) signature for overall survival in different subgroups of cohort 1. **(A)** MYCN not amplified. **(B)** MYCN amplified. **(C)** Favorable histology. **(D)** Unfavorable histology. **(E)** Differencing. **(F)** Poorly differentiated. **(G)** Hyperdiploid. **(H)** Diploid. **(I)** Low mitosis-karyorrhexis index (MKI). **(J)** Intermediate MKI. **(K)** High MKI. **(L)** Ganglioneuroblastoma. **(M)** Neuroblastoma. **(N)** Children's Oncology Group (COG) low risk. **(O)** COG high risk. **(P)** International Neuroblastoma Staging System (INSS) stage 4. **(Q)** Age > 18 months. The *p-*values were obtained using a Mantel log-rank test (two-sided).

Stratification survival analyses were performed in order to evaluate the prediction ability of the lncRNA signature in different clinical subgroups. The subgroups were classified according to MYCN amplification status (not amplified and amplified), histology subtype (favorable and unfavorable), differentiation status (differentiating and poorly differentiated), ploidy status (hyperdiploid and diploid), MKI status (low, intermediate and high), pathology subtype (ganglioneuroblastoma and NB), COG risk status (low and high), age status (age < 18 months and age > 18 months), and INSS stage. Within each subgroup, patients were classified into low-risk and high-risk subgroups on the basis of the same cutoff value from the entire cohort 1. Except for MYCN amplified subgroup, differentiating subgroup and high MKI subgroup, the lncRNA signature risk score significantly stratifies patients into two risk groups for OS in all of the other subgroups (Figure 8). Only one patient in stage 4s was classified as lncRNA high-risk group; thus, the Kaplan–Meier plot was not constructed. There is only one patient classified as stage 2, six patients classified as stage 3, and no patients classified as stage 1 in cohort 1. Thus, we did not conduct subgroup analysis for stage 1, stage 2, and stage 3.

### Gene Ontology Function Annotation and Gene Set Enrichment Analyses for the Prognostic Signatures

**Figure 9:**
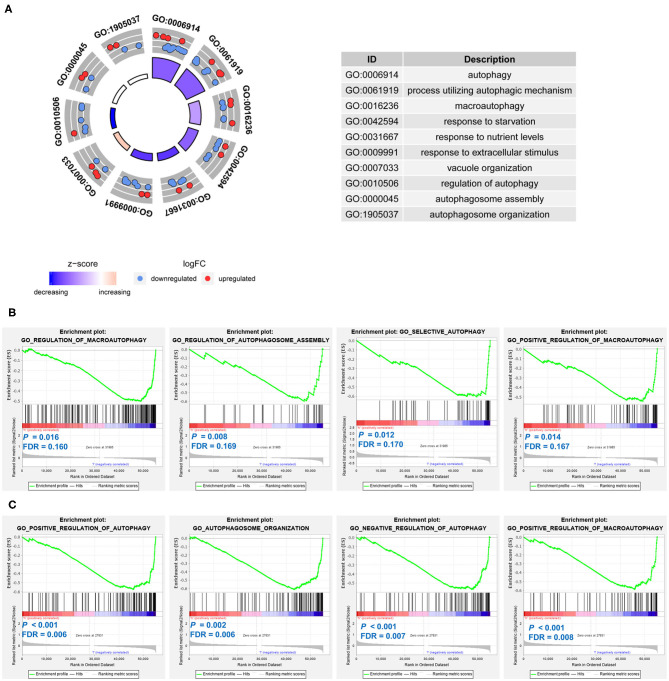
Function annotation and Gene Set Enrichment Analyses (GSEAs) of the prognostic signatures in neuroblastoma. **(A)** The circle plot of Gene Ontology (GO) function annotation for the differentially expressed and survival-related autophagy-related genes (ARGs). **(B)** Gene sets enriched in the low-risk group of the ARG signature. **(C)** Gene sets enriched in the low-risk group of the autophagy-related long non-coding RNA (lncRNA) signature.

The 21 differentially expressed and survival-related ARGs were put into GO functional annotation. The circle plot of GO revealed that autophagy biological processes (GO: 0006914 autophagy, GO: 0061919 process utilizing autophagic mechanism, GO: 0016236 macroautophagy, and GO: 0010506 regulation of autophagy) were down-regulated in stage 4 NB (Figure 9A).

GSEAs were also conducted to compare the difference between low-risk groups and high-risk groups. For both of the ARG signature and lncRNA signature, no autophagy-related gene set was enriched in the high-risk groups. Gene sets of GO regulation of macroautophagy, GO regulation of autophagosome assembly, GO selective autophagy, and GO positive regulation of macroautophagy were significantly enriched in the low-risk group of the ARG signature (Figure 9B). Gene sets of GO positive regulation of autophagy, GO autophagosome organization, GO negative regulation of autophagy, and GO positive regulation of macroautophagy were significantly enriched in the low-risk group of the lncRNA signature (Figure 9C).

### Genetic Alterations of the Genes in the Prognostic Signatures

The cBioportal platform was used to explore the genetic alterations of the nine ARGs and the seven lncRNAs in NB tumors (Supplementary Figure 5). The mutation data in 755 NB tissue samples and the somatic gene copy number data in 59 NB tissue samples were provided by cBioportal. The results showed that MYCN gene has somatic gene copy number alteration in about 19% of the NB tissue samples and has mutations in about 1.2% of the NB tissue samples. Only GABARAPL1 was found to have amplification in 1.7% of NB samples (Supplementary Figure 5A). SPNS1, DLC1, and ARNT were found to have missense mutation in 0.1% of NB samples (Supplementary Figure 5B). No gene alteration data were available for the lncRNA AC0022075.1 and AL356599.1. No somatic gene copy number alteration or mutation was detected for each of the other lncRNAs (Supplementary Figures 5C,D).

## Discussion

Autophagy is a highly conserved homeostatic pathway, which captures intracellular proteins and organelles and put them into degradation and recycling (21, 22). The role of autophagy in cancer is context dependent; in some models, autophagy could suppress cancer genesis, whereas some cancers are dependent on autophagy for survival (22). Some researchers reported the tumor-suppressive role of autophagy for NB. For example, one study reported that inhibition of cyclooxygenase-2 (COX-2) promotes 1-methyl-4-phenyl-1,2,3,6-tetrahydropyridine (MPTP)-induced autophagic cell death in human NB cell line SH-SY5Y (29); another study revealed that calcium/calmodulin-dependent protein kinase II (CAMK2) promotes autophagic degradation of inhibitor of differentiation 1/2 (Id-1/2) and then induces cell differentiation in NB (24). However, there are also studies that found the tumor-protective role of autophagy for NB. For example, rapid induction of ARG GABARAPL1 could promote NB cell survival before autophagy activation (25); autophagy was also found to be associated with chemoresistance of NB (26).

The association between ARGs and the spontaneous regression of NB is largely unknown. To our knowledge, this present study is the first with the purpose finding out ARGs associated with spontaneous regression of NB by combining both RNA-Seq and microarray data. Because spontaneous regression of NB is most prevalent in stage 4S NB patients, in this study, stage 4s tumors were used as surrogates to explore the underlining mechanisms responsible for the spontaneous regression of NB as many other investigators have done before. The dead cases in stage 4s were excluded to make it better to serve as surrogates. Actually, there were only two out of 21 stage 4s cases who died in cohort 1, five out of 54 stage 4s cases who died in cohort 2, and one out of 30 stage 4s cases who died in cohort 3 during more than 10-years of follow-up.

In this study, out of 233 ARGs in the Human Autophagy Database, a total of 48 ARGs were found to be differentially expressed between stage 4s and stage 4 NB samples, and 19 of these 48 ARGs were found to be significantly correlated with OS of NB patients. After LASSO Cox survival analysis, nine ARGs were found to have the best prognostic value and were used to construct an ARG prognostic signature. The ARG signature risk score successfully divided each of the cohorts into two different risk groups, with the low-risk group having good survival outcome and the high-risk group having bad survival outcome. The ARG signature also performed well in the subgroup survival analyses on the basis of different clinical risk factor stratifications. Multivariate survival analyses revealed that the prognostic role of this ARG signature is independent with other clinical risk factors. These results corroborate the role of autophagy in the genesis and progression of NB and suggest the use of this ARG signature as a risk factor for risk stratification.

Most NB patients in North America are treated according to the COG risk classification system. Based on MYCN amplification status, age at diagnosis, INSS stage, histopathology, and tumor cell ploidy, NB patients are stratified into low-, intermediate-, and high-risk groups according to the 2007 COG risk system (30, 31). The latest available data reveal that the 5-year OS rate was about 97% for COG low-risk NB patients (32); the 3-year OS rate was about 96% for COG intermediate-risk NB patients (33); and the OS rate for COG high-risk NB patients is only about 50% (31). In this study, although all patients in the COG low-risk subgroup were also classified as ARG signature low risk, the ARG signature significantly striated patients in the COG high-risk NBs into two risk groups. This suggests that the ARG signature risk score could improve the prognostic ability of COG risk classification system. We thus built a nomogram on the basis of the COG risk classification and the ARG signature risk score using the largest cohort (cohort 2, *n* = 498), which shows good accuracy for OS prediction. One drawback is that this dataset (cohort 2, GSE49710) only consists of COG low-risk and high-risk NB patients, whereas no NB patients were in the intermediated-risk group. In the TAGERT NBL cohort (*n* = 153), there are only several cases classified as COG intermediate risk, and we thus combined COG intermediate-risk and low-risk NBs together as one group during the analysis. However, because the OS rate of COG low-risk NB patients and COG intermediate-risk NB patients is similar (32, 33), we think that the influence of this drawback is limited.

The nine ARGs incorporated in the ARG signature include SPNS1 (sphingolipid transporter 1), TM9SF1 (transmembrane 9 superfamily member 1), WDR45B (WD repeat domain 45B), EIF4EBP1 (eukaryotic translation initiation factor 4E binding protein 1), GABARAPL1 (gamma-aminobutyric acid receptor-associated protein-like 1), ATG14 (autophagy related 14), ULK2 (unc-51 like kinase 2), DLC1 (DLC1 Rho GTPase activating protein), and ARNT (aryl hydrocarbon receptor nuclear translocator). Five (ATG4, ULK2, ARNT, GABARAPL1, and DLC1) of them are highly expressed in the low-risk group and are associated with good OS, whereas four (EIF4EBP1, WDR45B, SPNS1, and TM9SF1) of them are highly expressed in the high-risk group and are associated with bad OS.

The five ARGs that associated good survival of NB are all important positive regulators of autophagy. Two of them (ULK1 and ARNT) have been found to have important roles in regulating neuronal development: ULK1 is essential to mediate autophagy under nutrient-deficient conditions and regulate axon guidance in the developing forebrain of mouse via a non-canonical pathway (34, 35); ARNT is mostly expressed in neuronal cell types and play roles in regulating dendritic morphology and neuronal differentiation (36). Two of them (GABARAPL1 and DLC1) have been found to have a tumor-suppressive function: the GABARAPL1 protein could positively regulate ULK1 activity and autophagosome formation (37) and was also found to have a tumor-suppressive function in breast cancer cells (38, 39); DLC1 is involved in regulating autophagy and apoptosis and was found to be a potential tumor suppressor in many types of human cancers (40). ATG4 is the only protease functions as an important factor in the ATG8 conjugation system, and its activity is essential to autophagy (41). It has to be mentioned that one study revealed that rapid induction of GABARAPL1 promotes NB cell survival before autophagy activation (25), which is somewhat inconsistent with our findings as GABARAPL1 was found to be associated with good survival in our study. However, as is described in the literature, the protective role of GABARAPL1 for NB cell functions before autophagy activation (25).

The function of the four ARGs that associated bad survival of NB in our study has been reported as follows: EIF4EBP1 is a downstream target of mTOR signaling pathway and could inhibit autophagy initiation (42, 43); WDR45B was found to play an essential role in maintaining neural autophagy and neural homeostasis (44); SPNS1 was found to play an important role in orchestrating autolysosomal biogenesis and is critically linked to developmental senescence and survival (45); TM9SF1 was found to play important roles in inducing autophagy (46). Except for GABARAPL1, the roles of the other eight ARGs in NB genesis and progression have not been reported. The exact roles of these ARGs in NB and their underlining mechanisms need to be investigated by further studies.

LncRNAs are known as RNA transcripts longer than 200 nucleotides with no protein-coding capacity (47). LncRNAs are crucial players in various types of cancers including NB (48–52). In this study, we identified that the expression of 18 survival-related lncRNAs are correlated with the expression of the nine ARGs in the ARG signature. We termed these lncRNAs as autophagy-related lncRNAs. Seven autophagy-related lncRNAs were identified as having the best prognostic value by the LASSO Cox survival analyses. These seven autophagy-related lncRNAs were used to construct an autophagy-related lncRNA signature. The lncRNA signature risk score also successfully divided each of the cohorts into two different risk groups, with the low-risk groups having good survival outcome and the high-risk groups having bad survival outcome. The lncRNA signature performed well in the subgroup survival analyses on the basis of different clinical risk factor stratifications. Multivariate survival analyses revealed that the prognostic role of this lncRNA signature is also independent with other clinical risk factors. Different from the ARG signature, this lncRNA signature significantly stratified both COG low-risk NBs and COG high-risk NBs into two risk groups, indicating a somewhat better prediction accuracy. The *C*-index for the nomogram based on the lncRNA signature is a little higher than the *C*-index for the nomogram based on the ARG signature (0.85 vs. 0.84).

These seven lncRNAs incorporated in the lncRNA signature include FAM13A-AS1, SLX1A-SULT1A3, SNHG6, LINC001128, LINC00665, AL356599.1, and AC022075.1. Four (LINC01128, FAM13A-AS1, AL356599.1, and AC022075.1) of them are highly expressed in the low-risk group and are associated with good OS, whereas three (SLX1A-SULT1A3, LINC00665, and SNHG6) of them are highly expressed in the high-risk group and are associated with bad OS. The function of these lncRNAs is largely unknown. SNHG6 has been found to function by sponging microRNAs and to act as an oncogene in gastric cancer, colorectal cancer, and lung adenocarcinoma (53–55). LINC00128 could promote cervical cancer progression by binding with miR-383-5p and up-regulating Stratifin (56). The function of the other lncRNAs and their relation with cancer have not been reported in the literature. The relation of these lncRNA with autophagy, the role of their function in NB, and the underlying mechanisms still need to be clarified by further researches.

Investigation of the cBioportal platform discovered that GABARAPL1 has amplification in 1.7% of NB samples, whereas SPNS1, DLC1, and ARNT have missense mutation in 0.1% of NB samples. There is no somatic gene copy number alteration or mutation detected for these lncRNAs. It seems that genetic alterations play little roles in their differential expression in NB. However, this result should be interpreted with caution, as the number of NB cases providing genetic alteration information is limited. In addition, genetic alterations outside those genes might also be potential causes responsible for their altered expression. More studies are needed to figure out whether these ARGs and autophagy-related lncRNAs have genetic alterations in NB.

GO function annotation revealed that autophagy biological processes (GO: 0006914 autophagy, GO: 0061919 process utilizing autophagic mechanism, GO: 0016236 macroautophagy, and GO: 0010506 regulation of autophagy) were down-regulated in stage 4 NB. Consistent with the finding of GO function annotation, the GSEAs also revealed that autophagy gene sets were significantly enriched in the low-risk group: gene sets of GO regulation of macrophagy, GO regulation of autophagosome assembly, GO selective autophagy, and GO positive regulation of macroautophagy were significantly enriched in the low-risk group of the ARG signature, whereas gene sets of GO positive regulation of autophagy, GO autophagosome organization, GO negative regulation of autophagy, GO positive regulation of macroautophagy were significantly enriched in the low-risk group of the lncRNA signature. It is very interesting to find that no autophagy gene set is enriched in the high-risk groups. These results suggest that autophagy might mainly play a tumor-suppressive role in NB and might be associated with the spontaneous regression of NB. Undoubtedly, further investigations are needed to clarify how autophagy affects the process of spontaneous regression.

There are indeed some drawbacks in this study. Firstly, we did not perform *in vivo* or *in vitro* experimental studies to corroborate the findings of the present study. The exact roles of these identified ARGs or lncRNA in NB are largely unknown. Their underlining mechanisms responsible for NB progression or regression need to be clarified by further experimental studies. Secondly, spontaneous regression of NB did not occur in stage 4s tumors only, and not all cases in stage 4s underwent spontaneous regression. However, many other researchers have used stage 4s tumors as a surrogate. The dead cases in stage 4s NBs were also excluded in this study to make it better to serve as surrogates. Thirdly, the prognostic role of the signatures in some subgroups stratified by clinical risk factors showed no statistical significance. We think that the main reason is the low case number in these subgroups. Studies with larger sample size for these subgroups are needed. Despite these drawbacks, the combination of RNA-Seq data and microarray data, the large sample size of the three cohorts, and the validation of the findings by two independent cohorts all provide a high level of confidence.

In conclusion, we find that ARGs are differentially expressed between the stage 4 and stage 4s NB samples. The ARG prognostic signature has good performance in predicting OS of NB patients. The autophagy-related lncRNA signature also has good performance in predicting OS of NB patients. The prognostic value of both the ARG signature and lncRNA signature is independent of other clinical risk factors. The autophagy-related signatures have the potential to be used as risk factors for risk stratification of NB. Autophagy biological processes are significantly enriched in the low-risk groups and might mainly play a tumor-suppressive role in NB.

## Data Availability Statement

The dataset TARGET NBL was downloaded from https://portal.gdc.cancer.gov; the dataset GEO49710 was downloaded from https://www.ncbi.nlm.nih.gov/geo/query/acc.cgi?acc=GSE49710; the dataset E-MTAB-8248 was downloaded from https://www.ebi.ac.uk/arrayexpress/experiments/E-MTAB-8248/.

## Ethics Statement

Ethical review and approval was not required for the study on human participants in accordance with the local legislation and institutional requirements. Written informed consent from the participants' legal guardian/next of kin was not required to participate in this study in accordance with the national legislation and the institutional requirements.

## Author Contributions

This study was designed by XZ. Data were analyzed by XM, HL, EF, and XZ. The original manuscript was written by XM and HL. Supervision and manuscript revision were performed by XZ and JF. Funding was acquired by XZ and JF. All authors read and approved the final manuscript.

## Conflict of Interest

The authors declare that the research was conducted in the absence of any commercial or financial relationships that could be construed as a potential conflict of interest.
