# Supplementary material for: Comparison of Stage 4 and Stage 4s Neuroblastoma Identifies Autophagy-Related Gene and LncRNA Signatures Associated With Prognosis
Source: Front Oncol. 2020 Aug 19;10:1411. doi: 10.3389/fonc.2020.01411 (PMC7466450; doi:10.3389/fonc.2020.01411)
Supplement: Supplementary file 1 [file Data_Sheet_1.docx]

Supplementary Material

# Supplementary Data

## Supplementary Figures


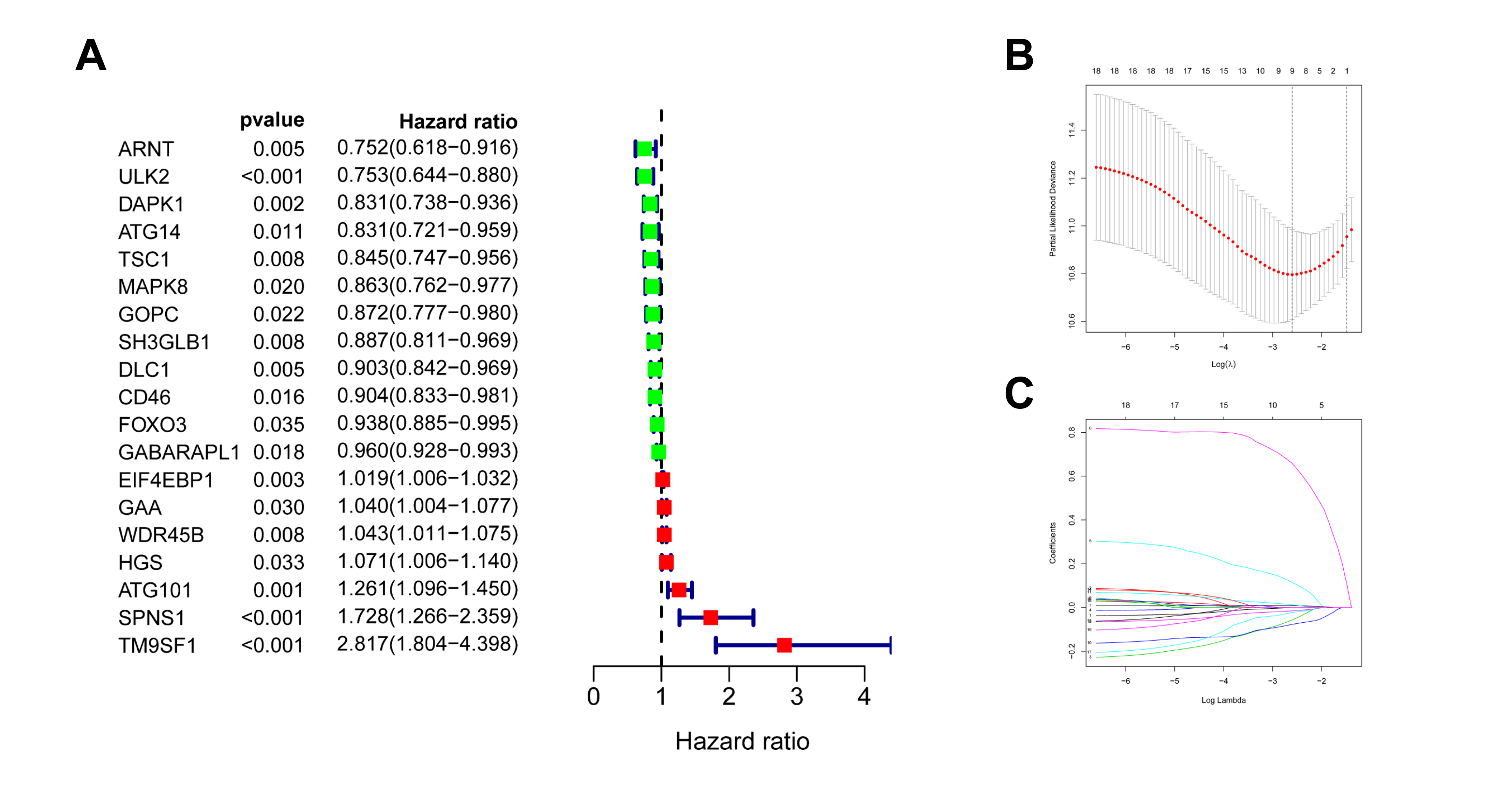


**Supplementary Figure 1.** Univariate Cox and LASSO Cox survival analyses for the ARGs in cohort 1. **(A)** Univariate Cox survival analyses for the differentially expressed and survival-related ARGs. **(B,C)** LASSO Cox analysis identified nine ARGs with the best prognostic value in cohort 1.


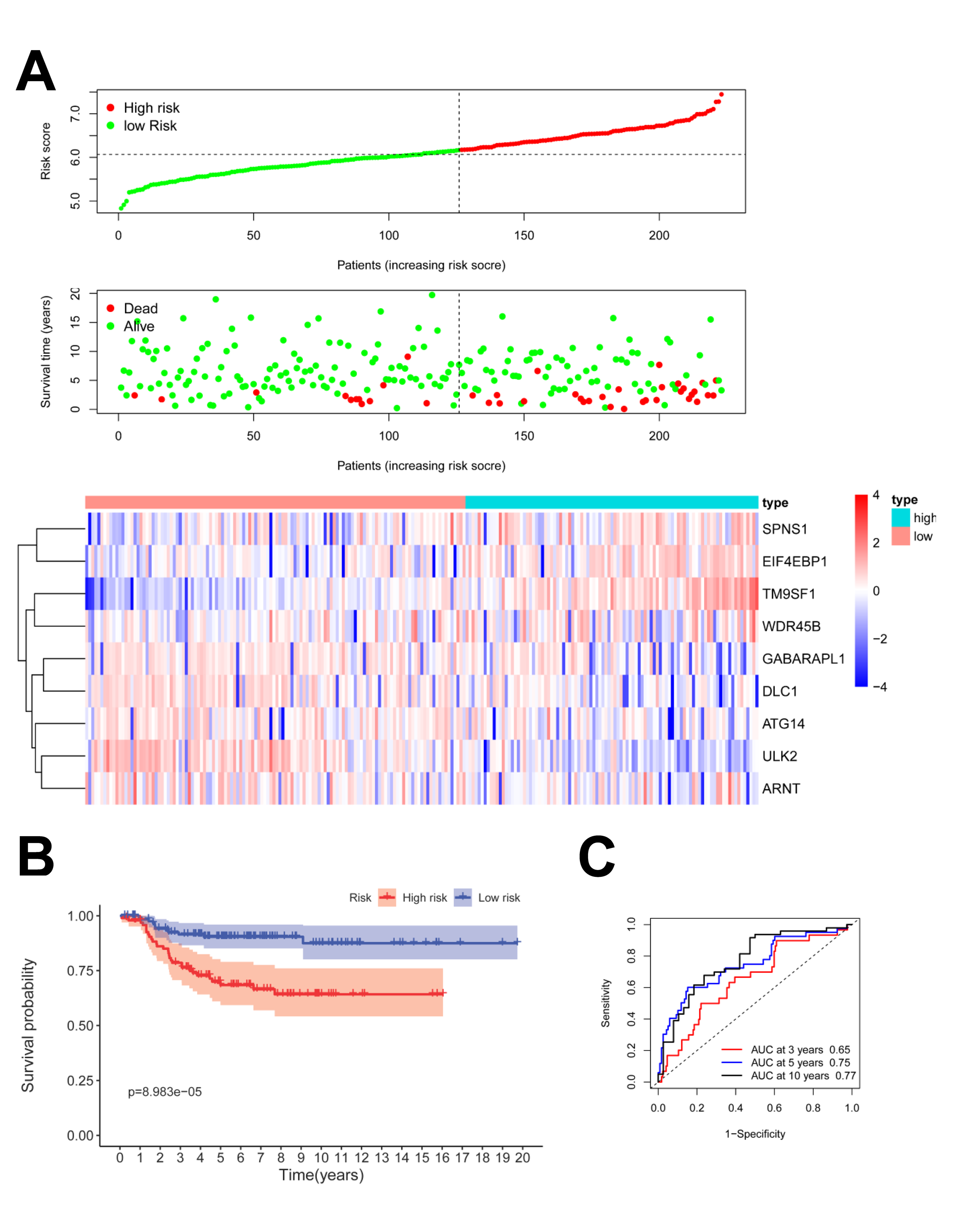


**Supplementary Figure 2.** The ARG signature risk score for neuroblastoma in cohort 3. (A) The distribution of risk scores, survival status of each patient, and heatmap of ARGs expression pattern in cohort 3. (B) Kalpan-Meier survival curve for OS of patients in the low risk group and high risk group for cohort 3. (C) Time-dependent ROC curves for the prognostic value of the ARG signature in cohort 3.


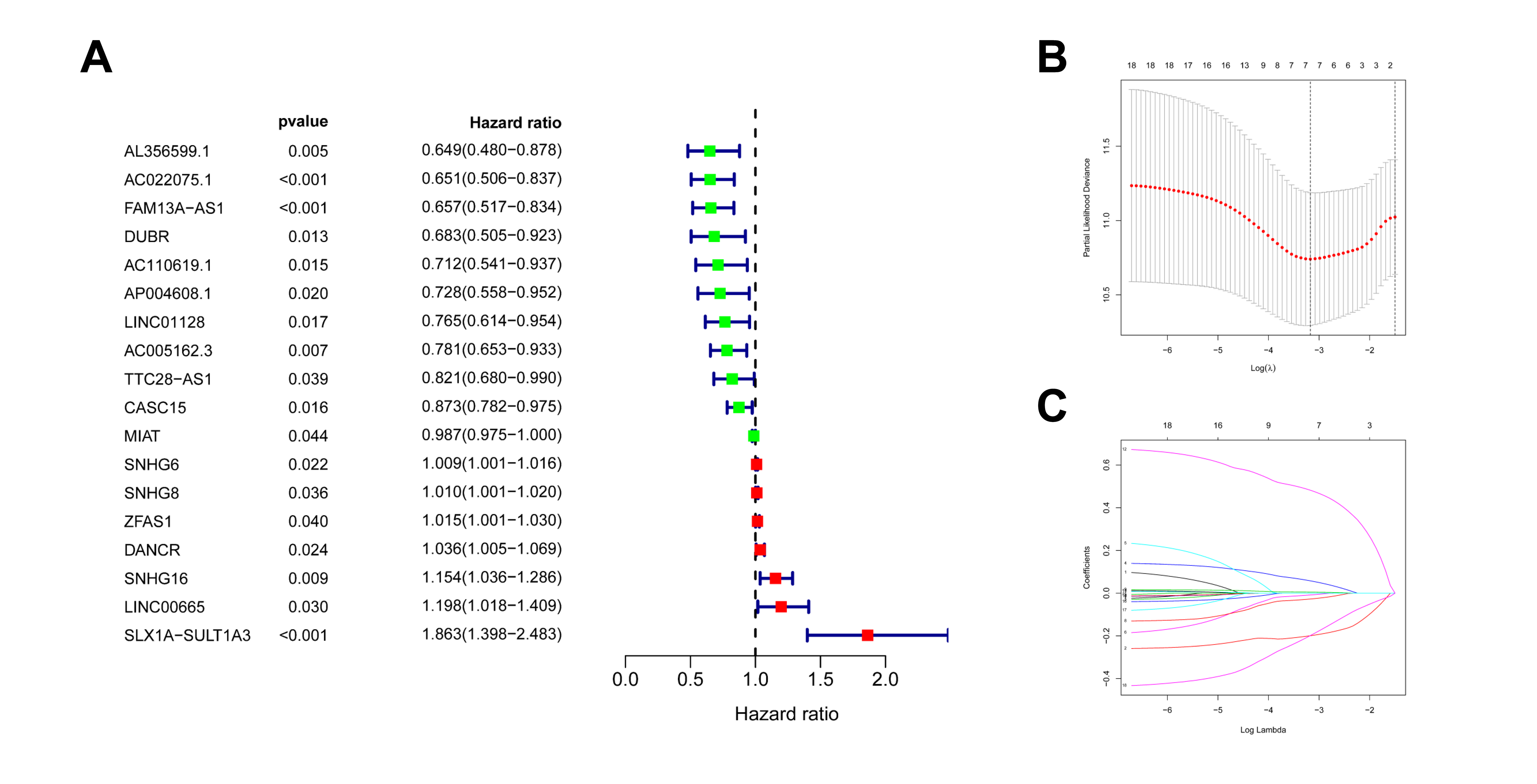


**Supplementary Figure 3.** Univariate Cox and LASSO Cox survival analyses for the autophagy-related lncRNAs in cohort 1. **(A)** Univariate Cox survival analyses for the survival-related lncRNAs. **(B,C)** LASSO Cox analysis identified seven lncRNAs with the best prognostic value in cohort 1.


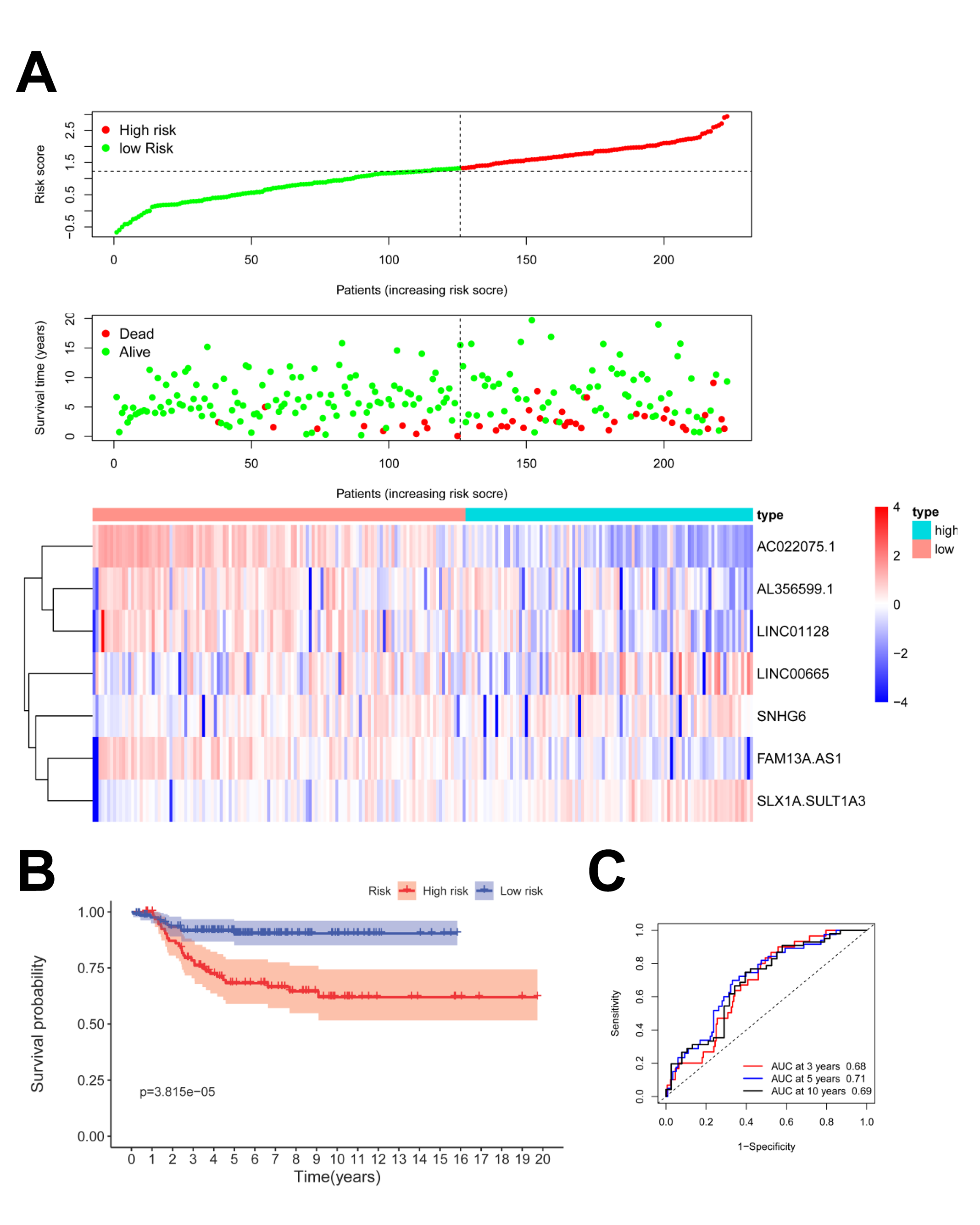


**Supplementary Figure 4.** The autophagy-related lncRNA signature risk score for neuroblastoma in cohort 3. (A) The distribution of risk scores, survival status of each patient, and heatmap of ARGs expression pattern in cohort 3. (B) Kaplan-Meier survival curve for OS of patients in the low risk group and high risk group for cohort 3. (C) Time-dependent ROC curves for the prognostic value of the lncRNA signature in cohort 3.


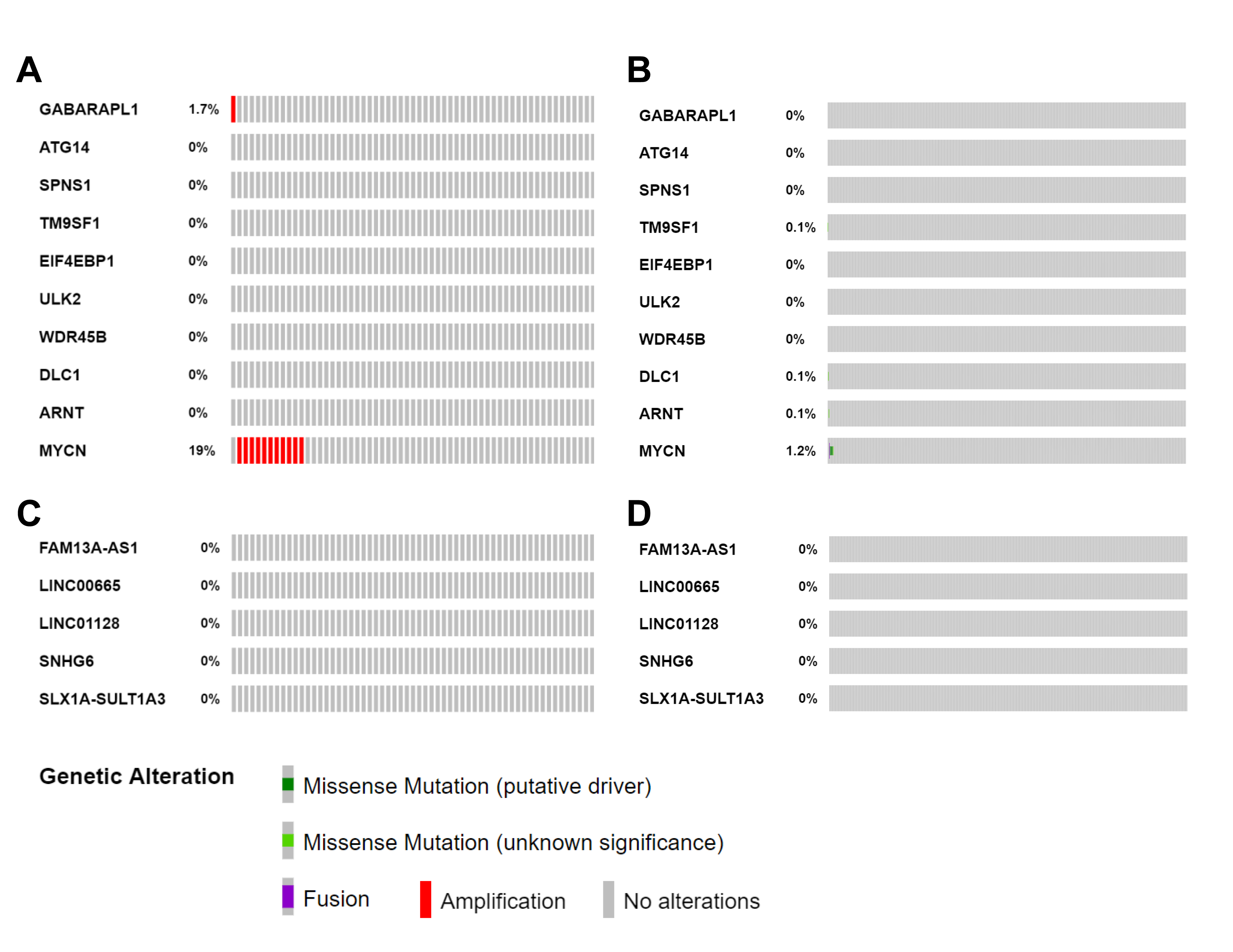


**Supplementary Figure 5.** Genetic alterations of the ARGs and lncRNAs in neuroblastoma. (A) Gene copy number alteration data of the ARGs in 59 NB cases. (B) Gene mutation data of the ARGs in 755 NB cases. (C) Gene copy number alteration data of the lncRNAs in 59 NB cases. (D) Gene mutation data of the lncRNAs in 755 NB cases.

## Supplementary Table

**Supplementary Table 1.** The clinical characteristics of the three cohorts.

|  | **No. of cohort 1 (%)** | **No. of cohort 2 (%)** | **No. of cohort 3 (%)** |
| --- | --- | --- | --- |
| **Age** |  |  |  |
| < 18 m | 29 (19.0%) | 300 (60.2%) | 103 (46.2%) |
| ≥ 18 m | 124 (81.0%) | 198 (39.8%) | 120 (53.8%) |
| **Gender** |  |  |  |
| Male | 89 (58.2%) | 287 (57.6%) | - |
| Female | 64 (41.8%) | 211 (42.4%) | - |
| **MYCN status** |  |  |  |
| Non-amplified | 121 (79.1%) | 401 (80.5%) | 176 (78.9%) |
| Amplified | 31 (20.3%) | 92 (18.5%) | 46 (20.6%) |
| **Risk** |  |  |  |
| Low | 27 (17.6%) | 322 (64.7%) | - |
| High | 126 (82.4%) | 176 (35.3%) | - |
| **INSS Stage** |  |  |  |
| 1 | 0 | 121 (24.3%) | 29 (13.0%) |
| 2 | 1 (0.7%) | 78 (15.7%) | 39 (17.5%) |
| 3 | 6 (3.9%) | 63 (12.7%) | 36 (16.1%) |
| 4 | 125 (81.7%) | 183 (36.7%) | 89 (39.9%) |
| 4S | 21 (13.7%) | 53 (10.6%) | 30 (13.5%) |
| **Vital status** |  |  |  |
| Dead | 77 (50.3%) | 105 (21.1%) | 42 (18.8%) |
| Alive | 76 (49.7%) | 393 (78.9%) | 181 (81.2%) |

**Supplementary Table 2.** The ARGs in the prognostic signature.

| **Gene Symbol** | **Official Full Name** | **Ensemble ID** | **Location** | **Log2FC** | **FDR** |
| --- | --- | --- | --- | --- | --- |
| ULK2 | unc-51 like autophagy activating kinase 2 | ENSG00000083290 | 17p11.2 | -1.02 | 1.16651E-06 |
| DLC1 | DLC1 Rho GTPase activating protein | ENSG00000164741 | 8p22 | -0.95 | 1.75394E-06 |
| GABARAPL1 | GABA type A receptor associated protein like 1 | ENSG00000139112 | 12p13.2 | -0.68 | 0.001463328 |
| ATG14 | autophagy related 14 | ENSG00000126775 | 14q22.3 | -0.56 | 0.000565624 |
| ARNT | aryl hydrocarbon receptor nuclear translocator | ENSG00000143437 | 1q21.3 | -0.52 | 2.03073E-05 |
| SPNS1 | sphingolipid transporter 1 (putative) | ENSG00000169682 | 16p11.2 | 0.52 | 0.005034856 |
| WDR45B | WD repeat domain 45B | ENSG00000141580 | 17q25.3 | 0.58 | 0.00016204 |
| EIF4EBP1 | eukaryotic translation initiation factor 4E binding protein 1 | ENSG00000187840 | 8p11.23 | 0.61 | 0.026991444 |
| TM9SF1 | transmembrane 9 superfamily member 1 | ENSG00000100926 | 14q12 | 0.81 | 1.09317E-06 |

**Supplementary Table 3.** The autophagy-related lncRNAs in the prognostic signature.

| **Gene Symbol** | **Official Full Name** | **Ensemble ID** | **Location** |
| --- | --- | --- | --- |
| LINC01128 | long intergenic non-protein coding RNA 1128 | ENSG00000228794 | 1p36.33 |
| AL356599.1 (FBXO30-DT) | FBXO30 divergent transcript | ENSG00000235652 | 6q24.3 |
| FAM13A-AS1 | FAM13A antisense RNA 1 | ENSG00000248019 | 4q22.1 |
| AC022075.1 (KLRK1-AS1) | KLRK1 antisense RNA 1 | ENSG00000245648 | 12p13.2 |
| SLX1A-SULT1A3 | SLX1A-SULT1A3 readthrough | ENSG00000213599 | 16p11.2 |
| LINC00665 | long intergenic non-protein coding RNA 665 | ENSG00000232677 | 19q13.12 |
| SNHG6 | small nucleolar RNA host gene 6 | ENSG00000245910 | 8q13.1 |
